# Pathological Findings of Nestling European Goldfinches (*Carduelis carduelis*) Co-Infected with *Klebsiella pneumoniae* and *Pseudomonas aeruginosa*

**DOI:** 10.3390/vetsci12090821

**Published:** 2025-08-27

**Authors:** Jessica Maria Abbate, Giulia D’Annunzio, Rosa Falleti, Claudio Gervasi, Valentina Ravaioli, Elisabetta Lilliu, Emma Santo, Elena Carra, Giovanni Tosi, Giovanni Lanteri

**Affiliations:** 1 Department of Chemical, Biological, Pharmaceutical and Environmental Sciences, University of Messina, V. le F. Stagno D’Alcontres 31, 98166 Messina, Italy; rosa.falleti@studenti.unime.it (R.F.); glanteri@unime.it (G.L.); 2 Experimental Zooprophylactic Institute of Lombardia and Emilia-Romagna (IZSLER) “Bruno Ubertini”, 41100 Modena, Italy; giulia.dannunzio@izsler.it (G.D.); emma.santo@izsler.it (E.S.); elena.carra@izsler.it (E.C.); 3 Experimental Zooprophylactic Institute of Lombardia and Emilia-Romagna (IZSLER) “Bruno Ubertini”, 47122 Forlì, Italy; valentina.ravaioli@izsler.it (V.R.); elisabetta.lilliu@izsler.it (E.L.); giovanni.tosi@izsler.it (G.T.)

**Keywords:** European Goldfinch, co-infection, *Klebsiella pneumoniae*, *Pseudomonas aeruginosa*, *ecfX*, *khe*, *rpoB*, 16S rRNA

## Abstract

Bacterial diseases are widespread in pet birds, posing a severe threat in nestlings and birds with weakened immune systems, often resulting in high mortality during the first days after hatching. This study aimed to describe the pathological findings of a natural bacterial co-infection with *Klebsiella pneumoniae* and *Pseudomonas aeruginosa* in nestling European goldfinches (*Carduelis carduelis*), as a cause of mortality after hatching. Eight nestlings kept in a breeding facility showed an inability to move, anorexia, and respiratory distress, and were found dead between 1 and 4 days of life. Gross pathological findings included diffuse pneumonia with edema and multifocal hemorrhages and occasionally hepatic necrosis. On histopathology, the main findings in all examined birds included severe, subacute bronchopneumonia and severe necrotizing hepatitis. Gram-negative bacilli were observed in parabronchial walls, within pulmonary arteries and surrounding hepatocellular necrotic foci. Lungs, livers and kidneys were sampled for bacteriological examination, resulting in two Gram-negative bacterial isolates. Four housekeeping genes (i.e., 16S rRNA; *rpoB*; *khe*; *ecfX*) were amplified and sequenced for bacterial identification at species level. Although *K. pneumoniae* and *P. aeruginosa* are common Gram-negative pathogens and are often co-isolated in human bacterial pneumonia, co-infection with these bacteria has not been documented in nestling goldfinches to date. Pathogen identification is essential for formulating a correct etiological diagnosis and further selecting the most appropriate therapeutic strategy.

## Simple Summary

This study describes the pathological findings associated with a natural bacterial co-infection caused by *Klebsiella pneumoniae* and *Pseudomonas aeruginosa* in nestling European goldfinches (*Carduelis carduelis*), which, to the best of the authors’ knowledge, has not been documented to date. Goldfinches were found dead between 1 and 4 days after hatching. On histopathology, pneumonia and hepatic necrosis with intralesional bacteria were the main pathological findings, identified as Gram-negative bacilli on Gram staining. Based on bacteriological and molecular investigations, *K. pneumoniae* and *P. aeruginosa* were identified and concomitant viral infections were excluded. Pathogen identification is certainly essential for formulating a correct etiological diagnosis and in choosing an appropriate therapeutic strategy. Furthermore, apparently healthy passerines, such as European goldfinches, may subclinically harbor pathogenic microorganisms of zoonotic interest.

## 1. Introduction

The European goldfinch (*Carduelis carduelis*) is a small passerine bird in the family *Fringillidae*, subfamily *Carduelinae*, commonly reared in captivity as an ornamental bird or for breeding and selection [1,2]. Goldfinches are often maintained in flocks, either with conspecifics in breeding facilities or with individuals of different species in mixed ornamental aviaries [3]. Although this is essential from an ethological perspective, as they are social birds, such close contact between birds provides an efficient mechanism for the transmission of various infectious agents [3,4].

Bacterial pathogens are widely distributed in One Health settings [5,6,7], including domestic birds, which can pose a significant threat, especially to animals with weakened immune systems and to nestlings, with high morbidity and mortality rates consistently observed in the first days post-hatching [8,9]. Respiratory and gastrointestinal tract infections are the most prevalent in bird chicks and, in severe cases, can rapidly progress to bacteriemia [8]. In most cases, clinically healthy immunocompetent adult birds are expected to play a central role in harboring and transmitting opportunistic and/or potentially pathogenic microorganisms in aviaries [10]. They may even serve as reservoirs of multidrug-resistant bacteria, posing a risk of transmission to humans, as well as serving as crucial indicator organisms in the cases of reverse zoonosis [3,11]. Pathogens can be transmitted vertically to offspring, leading to infections in developing embryos within the egg [12,13]. Transmission occurs during oviposition or shortly thereafter, through penetration of eggshells by maternal cloacal and fecal microorganisms spread throughout the nest environment. Furthermore, direct transfer after hatching can occur by parental oral microbes during feeding, which is also essential for the early assembly of the gut microbiota in nestling passerines [12,13].

The microbiota has a significant impact on health and disease, helping to prevent the colonization of pathogens and playing an essential role in the training and function of the host immune system in birds [14]. Accumulating evidence suggests that captivity influences the composition of the intestinal and respiratory microbiota in birds, as well as that the uncontrolled administration of antimicrobial drugs severely compromises its structure by promoting the overgrowth of common opportunistic bacteria [1,15,16,17]. The gut microbiota composition of domestic or captive avian species maintained on a restricted diet demonstrates an inadequate stress response to external stimuli, thus leading to an increase in the abundance of pathogenic microorganisms in their intestinal bacterial community [17]. Moreover, management practices have a significant impact on the occurrence of epidemics, as in the case of the uncontrolled introduction of new animals without quarantine, which represents a serious threat especially for nestlings or disease-weakened birds [4]. In passerine birds, the normal microbiota is typically composed of Gram-positive bacteria, whereas high proportions of Gram-negative bacteria are considered abnormal and associated with clinical disease, especially in most susceptible animals [1,18,19,20].

In particular, Gram-negative bacteria cause the majority of systemic infections in passerines, and *Klebsiella pneumoniae* and *Pseudomonas aeruginosa*, along with *Escherichia coli*, are common isolated bacteria [8,21]. As a well-known opportunistic bacterium, *Klebsiella pneumoniae* is frequently isolated from the feces and oropharynx of clinically healthy passerine and parrot species [22,23,24]. Nevertheless, *K. pneumoniae* acts as a causative agent of a variety of pathological conditions, including pneumonia, urinary tract infections, and sepsis, and is recognized as the causative agent of yolk sac infection and dead-in-shell among canary chicks [15,22,23,24,25,26]. Noteworthy, concomitant infectious or non-infectious diseases impact both the onset and the severity of *K. pneumoniae* infection [25]. Similarly, *P. aeruginosa* is considered a common avian pathogen, often causing disease as a secondary invader [15]. Generally, *P. aeruginosa* may not produce specific lesions in birds, especially in uncomplicated infections. Localized infections generally involve the upper respiratory tract in birds, although cases of septicemia, hemorrhagic enteritis, and mass mortality have been documented [15,27,28,29].

This study describes the pathological findings of *K. pneumoniae* infection associated with *P. aeruginosa* as causative agents responsible for mortality in nestling European goldfinches during the first week after hatching, in the absence of clinical disease in immunocompetent adults. To the authors’ knowledge, pathological findings associated with a natural co-infection caused by these two bacterial species have not been described in nestling goldfinches to date. Indeed, although bacterial infections represent a major cause of mortality in pet birds, with newly hatched chicks exhibiting high susceptibility, they remain poorly described in goldfinches.

## 2. Materials and Methods

### 2.1. Animals and Clinical Disease

An aviculturist reported the mortality of nestlings (*n* = 8) kept in two different cages (*n* = 5 from one cage; *n* = 3 from the other cage) during the first week after hatching. The deaths occurred over a 4-week period between April and May 2024, during which a total of twelve nestlings were born, of which 8 died during the first 1–4 days after hatching (8/12; 66.7% of mortality). Nestlings were kept in a breeding facility in Messina (Italy) of about 100 goldfinches, housed in pairs in cages located outside and exposed to natural daylight, and fed with commercial food. Several times a year, performing birds were taken to shows and competitions.

Immediately after hatching, the nestling finches showed lethargy and an inability to move, anorexia, respiratory distress, and abdominal distension and were found dead between 1 and 4 days after hatching. Neither clinical signs nor external lesions were observed in adult goldfinches kept in the same cages as the dead nestlings. Based on the clinical history symptoms, differential diagnoses included both bacterial causes and viral agents.

### 2.2. Necropsy and Histopathology

Postmortem examination was performed with the aid of a stereomicroscope on four of the eight dead nestling goldfinches, received at the Department of Chemical, Biological, Pharmaceutical and Environmental Sciences of the University of Messina. Tissue samples were sampled for bacteriology as described below, and stored frozen at −80 °C and fixed in 10% neutral buffered formalin, routinely processed and embedded in paraffin blocks. For histopathological examination, 3 µm thick, formalin-fixed paraffin-embedded (FFPE) tissue sections were stained with hematoxylin–eosin (HE) and reviewed by two board-certified pathologists (J.M.A.; G.D.). Gram staining was also performed using 3 µm thick tissue sections.

### 2.3. Bacteriological Investigation

Lungs, livers and kidneys of a nestling were sampled for bacteriological examination by incising them with a sterile scalpel and placing a sterile swab in the incision. Swabs were rolled onto 5% sheep blood agar plates (Biolife, Milan, Italy) and subsequently incubated aerobically for 72 h at 28 °C. Colonies were subcultured individually multiple times onto plates with the same medium, incubated each time at 28 °C for 48 h until pure cultures derived from a single colony were obtained. Finally, pure culture was smeared on a glass slide and subjected to Gram staining.

### 2.4. Molecular Identification of the Bacterial Colonies

Genomic DNA was extracted from pure bacterial cultures as described below. Briefly, liquid cultures were prepared using the two different bacterial isolates obtained at a concentration of 2 × 10^6^ bacterial cells and then centrifuged at 5000× *g* for 10 min at 4 °C. The resulting pellet was used for genomic DNA extraction using the GeneJET Genomic DNA Purification Kit (Thermo Scientific, Milan, Italy) following the manufacturer’s instructions.

The 16S rRNA, *ecfX*, *khe* and *rpoB* gene sequences for the bacterial isolates were determined by PCR and using the primers listed in Table 1.

PCR was performed using GoTaq^®^ Colorless Master Mix (Promega, Madison, WI, USA) in a final reaction volume of 25 µL, using 12.5 µL of GoTaq^®^ Colorless Master Mix, 1 µL of each primer (10 µM) and 1 µL of extracted DNA (<250 ng/µL). The cycling conditions for 16S rRNA were set as recently described by our research group [33]. Briefly, the PCR protocol for other primers included an initial denaturation at 95 °C for 5 min, followed by 30 cycles of denaturation at 94 °C for 30 s (35 cycles at 94 °C for 45 s for *ecfX*), annealing at 58 °C for 45 s for *ecfX*, 68 °C for 40 s for *khe*, 50 °C for 40 s for *rpoB*, elongation at 72 °C for 60 s and a final extension step at 72 °C for 5 min [30,31,32]. For all amplifications, negative controls were included omitting the DNA. Electrophoresis of PCR products was performed in 1.5% (*w/v*) agarose gel and amplicons were visualized under ultraviolet (UV) light. The nucleic acid concentration and purity were measured using a Nanodrop Spectrophotometer (NanoPhotometer N50, IMPLEN, Westlake Village, CA, USA).

DNA sequencing of the purified PCR products was performed by Genechron (Rome, Italy) using the same forward and reverse primers used for the PCR reactions. Sequence alignments were performed using the ClustalW algorithm (https://www.genome.jp/tools-bin/clustalw; accessed on 24 November 2024). Sequences were analyzed with the Basic Local Alignment Search Tool (BLAST, http://blast.ncbi.nlm.nih.gov/; accessed on 24 November 2024) to search for similarities against the National Centre for Biotechnology Information (NCBI) database (https://blast.ncbi.nlm.nih.gov/Blast.cgi; accessed on 24 November 2024) and calculate the statistical significance of the matches.

### 2.5. PCR-Based Diagnostic for Viral Infectious Agents

FFPE tissue sections from all organs were placed in single microcentrifuge tubes and pretreated by adding 1 mL of xylene, vortexed vigorously, incubated at room temperature for 30 min, and centrifuged at 11,000× *g* for 3 min, and supernatants were then pipetted off. Two washes with ethanol (96–100%) were then performed by adding 1 mL to each tube, closing, and mixing by inverting several times, and then ethanol was pipetted off and the open tubes were incubated at 37 °C for about 15 min, until the alcohol had completely evaporated. Then, each sample was pre-lysed by adding 350 µL of Lysis Buffer T1 (Macherey-Nagel, Duren, Germany) and 50 µL of Proteinase K (Qiagen, Hilden, Germany) to each tube, followed by an incubation step at 56 °C overnight. Samples were then ready for automated purification of viral nucleic acids using the QIAsymphony DSP Virus/Pathogen Kit (Qiagen, Hilden, Germany) in combination with the QIAsymphony SP instrument, according to the manufacturer’s instructions. Extracts were eluted in 60 µL of Elution Buffer.

Amplification of viral nucleic acids was performed using different amplification kits and primer/probe sets depending on the virus investigated (Table 2).

*NewCastle Disease Virus*: Real-time RT-PCR assay was performed with the VetMAX-Plus Multiplex One-Step RT-PCR Kit (Applied Biosystems, Foster City, CA, USA) according to the manufacturer’s instructions. The amplification reaction was carried out in a final volume of 25 µL according to the listed thermal cycling conditions: 10 min at 48 °C, 10 min at 95 °C followed by 40 denaturation cycles at 95 °C for 15 s and annealing/extension at 50 °C for 45 s [34].

*Avian Bornavirus*: Real-time RT-PCR assay was performed with the VetMAX-Plus Multiplex One-Step RT-PCR Kit (Applied Biosystems, Foster City, CA, USA) according to the manufacturer’s instructions. The amplification reaction was carried out in a final volume of 25 µL according to the listed thermal cycling conditions: 20 min at 50 °C, 5 min at 95 °C followed by 40 denaturation cycles at 95 °C for 15 s and annealing/extension at 53 °C for 60 s [35].

*Canary Polyomavirus*: A nested PCR was performed using the GoTaq G2 Hot Start Master Mix 2x (Promega, Madison, WI, USA) according to the manufacturer’s instructions in a final volume of 25 µL. The first cycling protocol included 5 min of incubation at 95 °C, followed by 45 cycles each of 94 °C for 30 s, 52.2 °C for 1 min and 72 °C for 1 min, and 72 °C for 5 min. For nested PCR, 4 µL of the first PCR product was used as template in a similar reaction at 95 °C for 5 min, 45 cycles of 94 °C for 30 s, 62 °C for 30 s and 72 °C for 30 s, and 72 °C for 5 min [36].

*Canary Circovirus*: End-point PCR was performed using the GoTaq G2 Hot Start Master Mix 2x (Promega, Madison, WI, USA) according to the manufacturer’s instructions. The amplification reaction was carried out in a final volume of 25 µL according to the listed thermal cycling conditions, denaturation step at 94 °C for 2 min and by 40 cycles of PCR, each consisting of 30 s at 94 °C, 30 s at 52 °C and 1 min at 68 °C. Reactions were completed with a final elongation step for 5 min at 68 °C [37].

The PCR products were electrophoresed in a 2% Tris–acetate-EDTA-agarose gel and stained with GelRed Nucleic Acid Stain (BIOTIUM). UV trans-illumination was used to visualize the DNA bands. The size of DNA fragments was estimated by comparison with the 100 bp DNA Ladder (Invitrogen, Waltham, MA, USA).

## 3. Results

### 3.1. Gross Pathology

**Figure 1:**
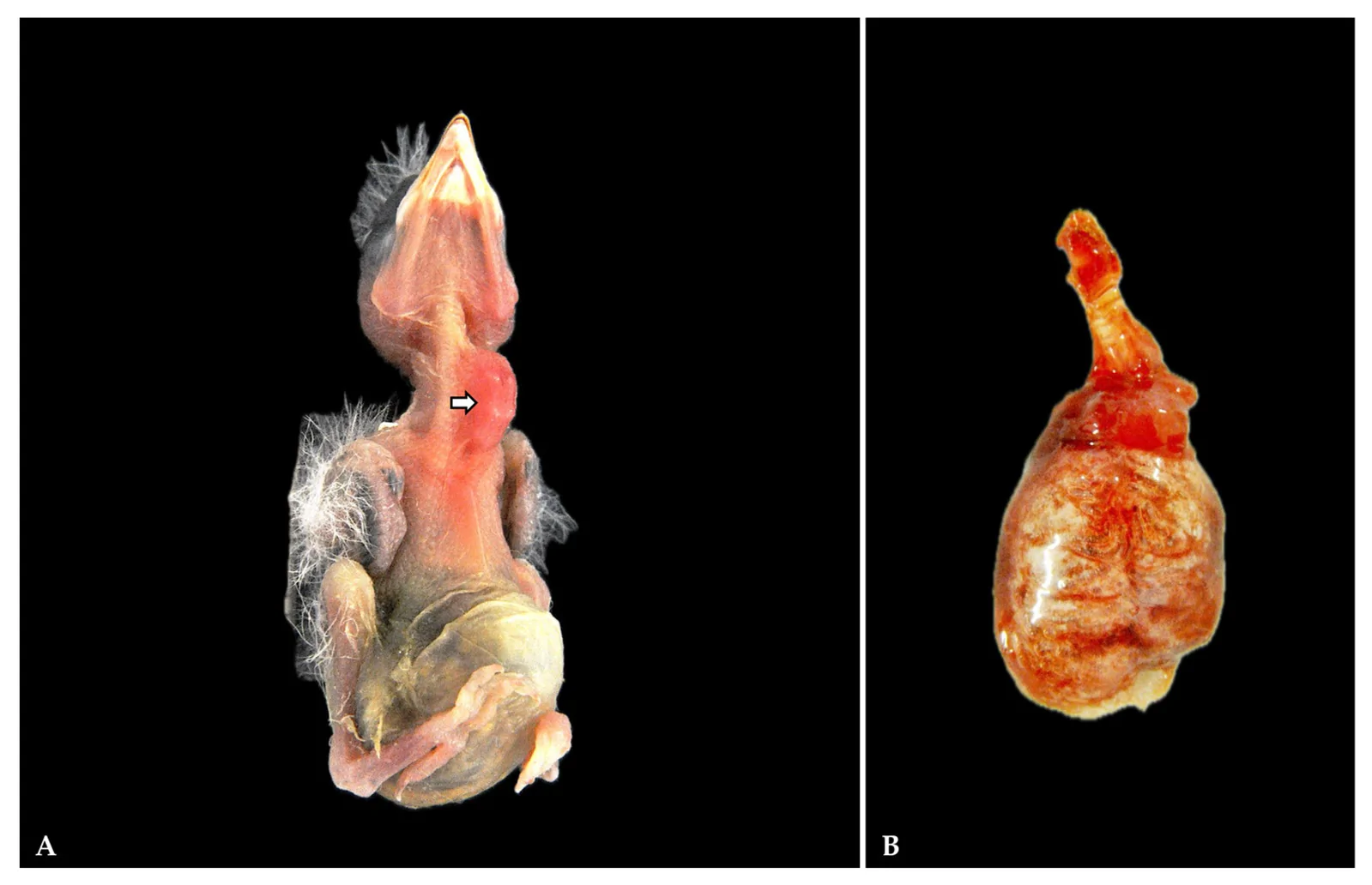
Gross pathology. (**A**) The nestling goldfinches were in poor body condition, with a distended crop (arrow) and coelom. (**B**) Lung. Diffuse reddish coloration, edema, and multifocal hemorrhages were observed in the lungs of all examined goldfinches.

Nestling goldfinches (*n* = 4), 7.50–8.20 cm in length and weighing 2.5–3.9 g, were in poor body condition with no fat tissue reserves. At gross examination, common findings were the presence of distended crop (*n* = 2; 50%) and coelom (*n* = 4; 100%) (Figure 1A).

On gross examination, livers showed minimal or no gross change (2/2; 50%) and were occasionally slightly enlarged with rounded edges (1/4; 25%), or contained randomly distributed, multifocal areas of discoloration, suggestive of necrosis (1/4; 25%).

In all examined nestlings (4/4; 100%), lungs were diffusely reddish in color, showing multifocal hemorrhages and edema (Figure 1B). In one goldfinch (1/4; 25%), a focal extensive area of epicardial hemorrhage was observed.

### 3.2. Histopathology

#### 3.2.1. Respiratory Tract

**Figure 2:**
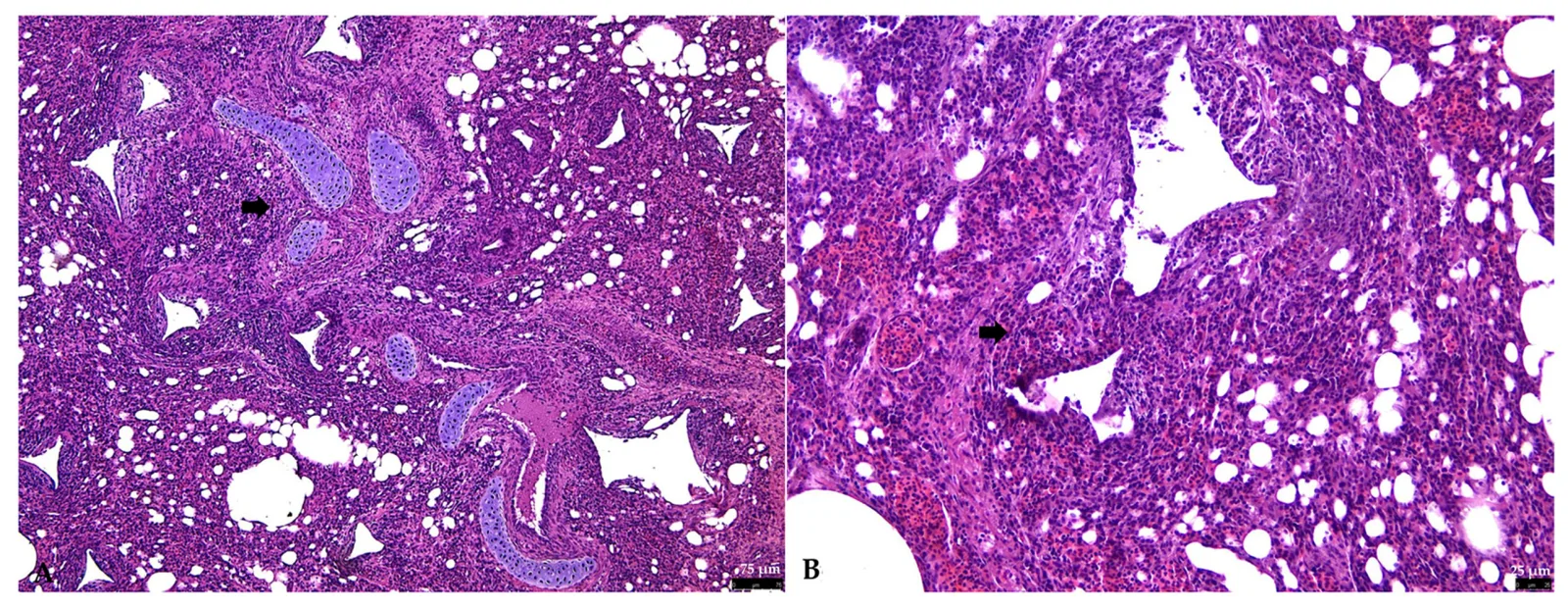
Representative histopathology of lung. An abundant inflammatory infiltrate expands the parabronchial walls, multifocally obscuring the secondary bronchi and respiratory atria (arrow). Hematoxylin–eosin (HE): (**A**) magnification 100×; (**B**) magnification 200×.

In all nestlings (*n* = 4; 100%), the lungs were characterized by moderate-to-severe inflammation, involving 70–80% of the pulmonary parenchyma multifocally, expanding and effacing the parabronchial walls and commonly obscuring the secondary bronchi and respiratory atria (Figure 2).

**Figure 3:**
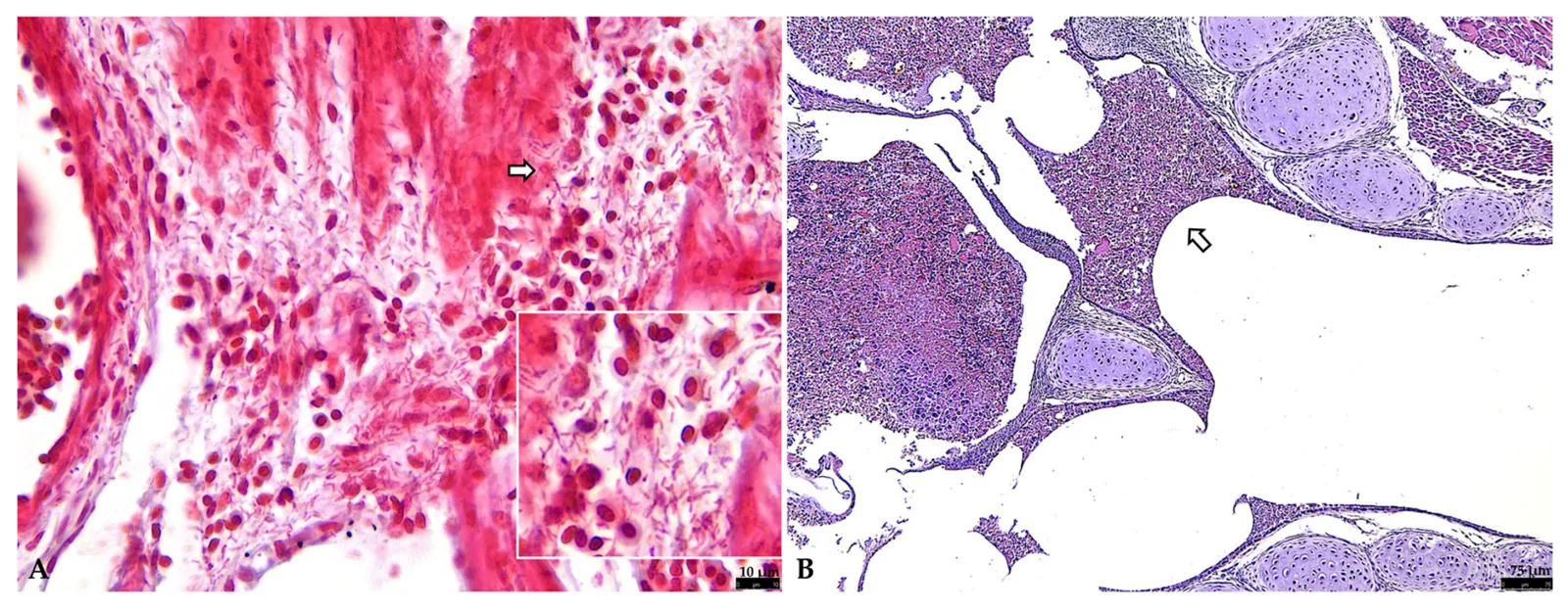
(**A**) Lung. Numerous aggregates of Gram-negative bacilli (arrow) are evident in parabronchial walls and within the arterial lumen. Inset shows bacterial aggregates in the lung. Gram stain, magnification 630×; (**B**) Syringes and trachea. Mucous exudate mixed with necrotic cellular debris, inflammatory cells, extravasated red blood cells within the lumen (arrow). Hematoxylin–eosin (HE), magnification 100×.

The inflammatory infiltrate consisted of numerous lymphocytes, moderate numbers of plasma cells and macrophages, and occasional viable and necrotic heterophils. Inflammatory cells were multifocally admixed with necrotic epithelial cells and/or embedded in eosinophilic fibrillar material (fibrin). Multifocal hemorrhages of variable size were also present, along with hemosiderin-laden macrophages. Multifocally, bronchial lumina and respiratory atria were obscured and filled with mucous exudate and/or edema with scattered sloughed necrotic cellular debris. Blood vessels in the parabronchial septa were diffusely hyperemic. Scattered aggregates of bacilli (2–3 micron long) were observed throughout the parenchyma and often within the lumen of the pulmonary arteries and confirmed as Gram-negative bacilli on Gram staining in all nestlings (Figure 3A). The definitive diagnosis for all nestlings was subacute, severe, multifocal-to-coalescing lymphocytic–plasmacytic bronchopneumonia.

A moderate amount of mucous exudate mixed with necrotic cellular debris, inflammatory cells, extravasated red blood cells, and scattered basophilic bacteria (bacilli) was observed within the trachea and syringes of all nestlings examined (Figure 3B).

The nasal cavity epithelium was necrotic and sloughed into the lumen, which contained abundant mucus mixed with necrotic debris and inflammatory cells.

#### 3.2.2. Liver

**Figure 4:**
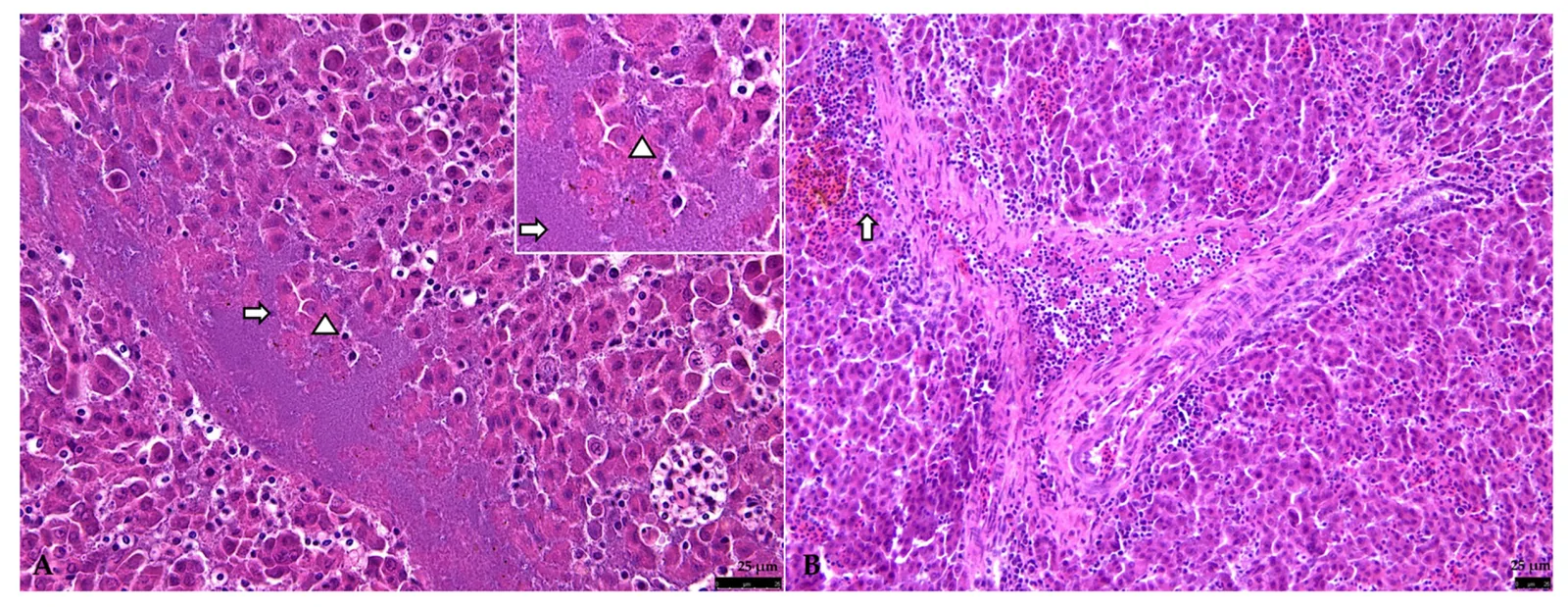
Representative histopathology of the liver. (**A**) Disruption of the hepatocyte cords with extensive areas of lytic necrosis and dystrophic mineralization (arrow), and scattered aggregates of basophilic bacteria (bacilli) (triangle). Inset shows dystrophic mineralization (arrow) and intralesional bacilli (triangle). Hematoxylin–eosin (HE), magnification 400×. (**B**) Multifocal infiltration of inflammatory cells in periportal area with multifocal hemorrhages between necrotic hepatocytes (arrow). Hematoxylin–eosin (HE), magnification 200×.

Multifocally, random foci of severe hepatocellular necrosis associated with abundant basophilic bacilli (2–3 micron long) were observed in all examined nestling goldfinches (*n* = 4; 100%). Specifically, 30–40% of the hepatic parenchyma was characterized by foci of lytic necrosis with occasional concurrent areas of mineralization (dystrophic mineralization), with disruption of the hepatocyte cords and loss of normal hepatic architecture. Moderate numbers of lymphocytes, scattered plasma cells and macrophages expanded the portal tracts and periportal areas. Sinusoids were diffusely engorged by erythrocytes. Multifocally, hepatic hemorrhages with scattered concurrent hemosiderin-laden macrophages were observed (Figure 4).

**Figure 5:**
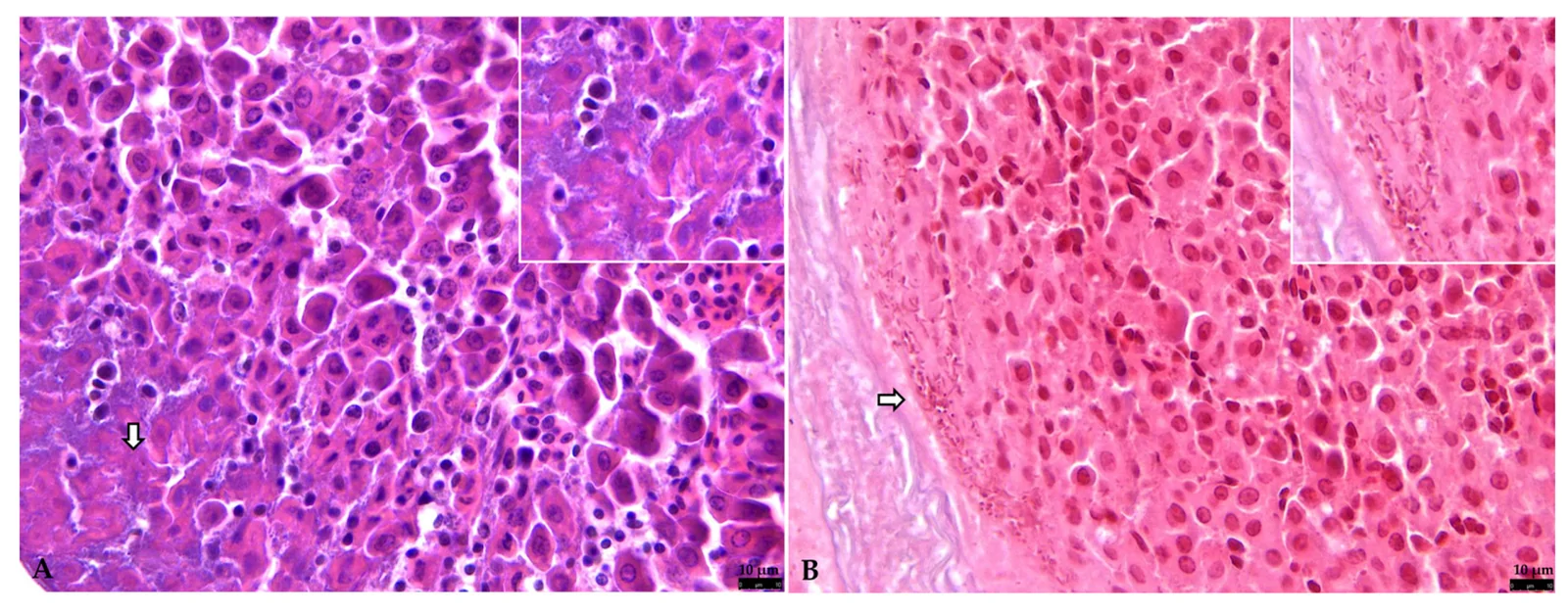
Representative histopathology and histochemistry of the liver. (**A**) Aggregates of basophilic bacteria (bacilli) (arrow). Inset shows bacilli in the liver. Hematoxylin–eosin (HE), magnification 630×. (**B**) Gram-negative bacilli (arrow). Inset shows Gram-negative bacilli in areas of extensive hepatocellular necrosis. Gram staining, magnification 630x.

Multifocal aggregates of abundant basophilic bacilli were observed within and surrounding the hepatocellular necrotic foci (Figure 5A) and confirmed as Gram-negative bacilli on Gram stain (Figure 5B).

#### 3.2.3. Other Organs

**Figure 6:**
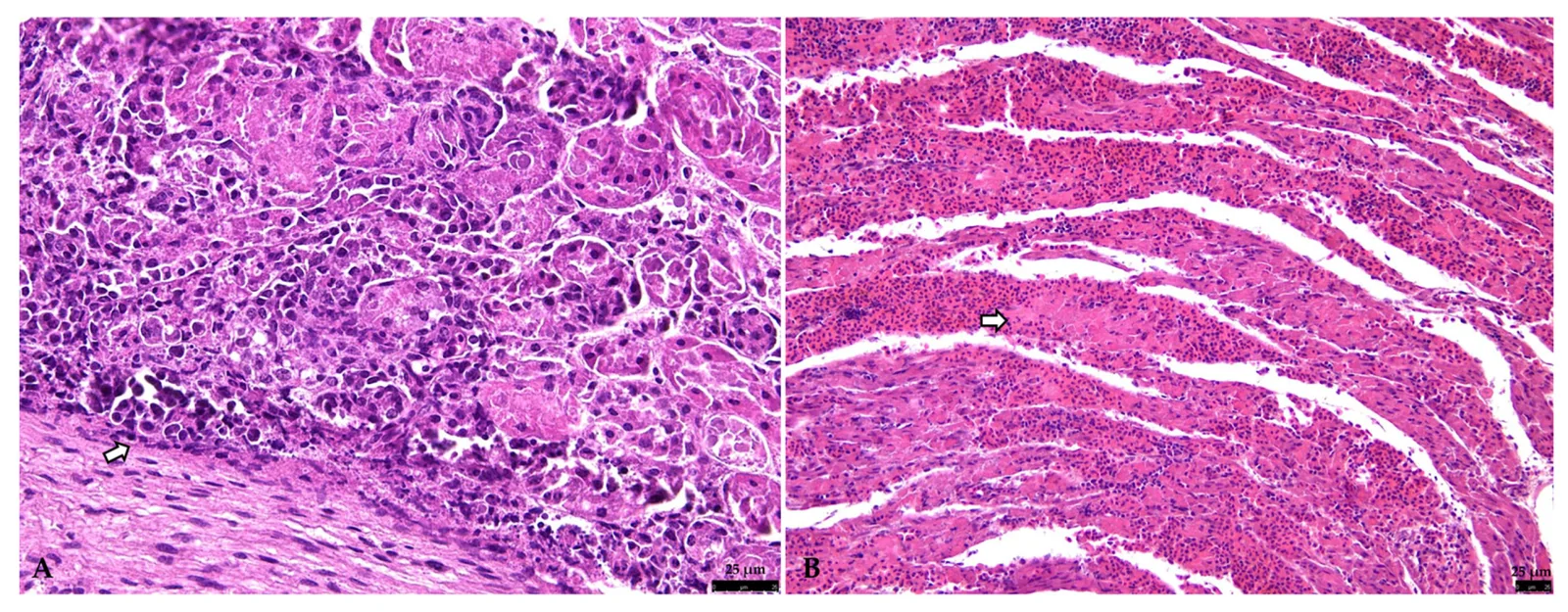
Histopathology of other organs. (**A**) Kidney. Multifocal interstitial, lymphocytic-plasmacytic nephritis (arrow), together with epithelial necrosis of proximal convoluted tubules. Hematoxylin eosin, magnification 400×. (**B**) Heart. Focal area of myocardial hemorrhages, multifocal necrosis of cardiomyocytes and extravasation of inflammatory cells (arrow). Hematoxylin–eosin (HE), magnification 100×.

Mild multifocal interstitial nephritis was observed in two out of the four nestlings examined, with infiltration of small numbers of lymphocytes, viable and necrotic heterophils, and macrophages. Multifocally, the proximal convoluted tubules were characterized by necrotic epithelial cells, with loss of brush border and karyolitic or pyknotic nuclei, and cells were often sloughed into the lumen with occasional dystrophic calcification (Figure 6A). In a single finch, a focally extensive area of myocardial hemorrhages was observed, together with segmental, coagulative and lytic necrosis of cardiomyocytes and multifocal extravasation of inflammatory cells (Figure 6B). No other histopathological lesions were observed in other organs.

### 3.3. Bacterial Identification

Two different Gram-negative bacterial isolates were obtained from sampled organs (lung, liver, and kidney). Nucleotide sequences of the amplified PCR products obtained from both colonies were identical to their respective biological replicates. BLAST analysis (NCBI) of 16S rRNA gene sequences obtained from the two bacterial isolates showed a similarity of 99.22% with reference sequences of *Klebsiella pneumoniae* (Query Cover 99%, E value 0) and 99.81% with sequences of *Pseudomonas aeruginosa* (Query Cover 100%, E value 0), respectively. Representative 16S rRNA sequences were submitted to GenBank (accession numbers: PP980564; PP980565) (File S1).

Amplification and sequencing of *ecfX*, *rpoB* and *khe* genes were performed to identify *P. aeruginosa* and *K. pneumoniae* to the species level.

The different primers used in this study generated amplicons of 500 bp for *ecfX*, 1000 bp for *rpoB*, and 430 bp for *khe*. BLAST analysis of the sequences obtained from these amplicons confirmed the identification of bacterial isolates with high similarity to reference strains.

Amplification of the *ecfX* gene yielded a 500 bp fragment that, after sequencing and BLAST analysis, showed 100% identity with *P. aeruginosa* (Query Cover: 100%, E-value: 0). Similarly, the *rpoB* gene amplification generated a 1000 bp amplicon, which showed 100% similarity to *K. pneumoniae* (Query Cover: 100%, E-value: 0). The *khe* gene amplification produced a 430 bp fragment, further confirming the identification of *K. pneumoniae*, also with 100% sequence identity (Query Cover: 100%, E-value: 0). Representative sequences were submitted to GenBank under accession numbers PV578954, PV578955 and PV578956.

### 3.4. Molecular-Based Diagnostic for Viral Agents

None of the investigated viral pathogens were detected.

## 4. Discussion

This study reports the pathological findings of a natural co-infection with *Klebsiella pneumoniae* and *Pseudomonas aeruginosa*, as a cause of mortality in finches during the first week after hatching. Although these bacteria are common Gram-negative pathogens associated with bacterial pneumonia, often isolated simultaneously in humans [38], we have not recovered published cases of *K. pneumoniae*- and *P. aeruginosa*-associated pneumonia in goldfinches. A contagious disease was suspected based on the clinical history and mortality of nestlings housed on the same breeding facility and showing similar, although nonspecific, gross pathological findings. Both bacterial and viral diseases were included in differential diagnoses, and based on histopathology, microbiology and molecular investigations, a mixed bacterial infection caused by two widespread, opportunistic Gram-negative pathogens was confirmed. *K. pneumoniae* is generally considered a primary pathogen, particularly in some finches [21]. Conversely, *P. aeruginosa*, a common avian pathogen, often causes disease as a secondary invader in birds that are already weakened or compromised by other diseases, such as viral infections or nutritional deficiencies [15,39].

Of note, co-infection with *P. aeruginosa* has been shown to increase *K. pneumoniae* proliferation in the lung of mice and its subsequent bacteriemia, resulting in increased lethality compared to *K. pneumoniae* infection alone [40]. Similarly, in human patients, co-infection with these two bacteria was identified as the strongest independent predictor for 90-day mortality [41], and the common co-isolation of *K. pneumoniae* and *P. aeruginosa* from human lungs highlights the natural propensity of these bacteria to coexist [38]. Several microorganisms in nature typically exist in multispecies biofilms in which cooperative or competitive interactions between species define the composition and spatial organization of member species [42], as well as resilience of the mixed-species biofilm [43]. Of note, bacterial pathogens such as *K. pneumoniae* and *P. aeruginosa* exploit quorum sensing (QS) cell-to-cell communication to coordinate the expression of diverse virulence factors and associated behaviors such as biofilm formation [44]. The expression of QS-regulated virulence factors coordinates the bacterial attack against the host, maximizing the likelihood of establishing infections as well as bacteriemia, thus enhancing the overall pathogenicity of the pathogen [44]. In polymicrobial infections, QS represents a complex and dynamic interplay between different bacterial species, and QS-mediated interactions facilitate both cooperative and competitive behaviors [45]. Of note, a cooperative interaction was found between *K. pneumoniae* and *P. aeruginosa* [42]. Therefore, even in finches examined here, it is plausible that co-infection with *P. aeruginosa* exacerbated pneumonia caused primarily by *K. pneumoniae*, leading to subsequent bacteriemia and mortality.

Bacterial infections are common in domestic birds and can lead to death, especially in immunodepressed or weakened chicks during the first days after hatching, due to various pathological syndromes [9]. Their transmission can be vertical, with infections occurring during egg development in the reproductive tract and resulting in early mortality [12,13]. Moreover, opportunistic and potentially pathogenic bacteria may be part of maternal cloacal and fecal microbes and can directly enter the eggshell during the oviposition or incubation period as they are smeared in the nest, causing infection of hatching eggs and newly hatched chicks [12,13,46,47]. Furthermore, microbes from the oral cavity and crop of finches are normally transferred through feeding during the first days of life, and this route is essential in the early establishment of the gut microbiota in passerine chicks [48]. Interestingly, *K. pneumoniae* is often isolated from oropharyngeal swabs and fecal material of clinically healthy passerines and psittacines, although this enterobacterium does not belong to the normal gut flora of granivorous pet birds including finches [23,49,50]. *K. pneumoniae* is capable of spreading in the environment and becoming ubiquitous under appropriate conditions. In fact, in birds, the development of infection with intestinal or even extraintestinal disease typically requires the presence of predisposing factors [23,49,50]. Similarly, *P. aeruginosa* represents the second most frequent isolated species among *Pseudomonadaceae* in several free-living bird species, as part of the pharyngeal and cloacal bacterial microbiota [51], while food and water contamination represents the main source of *P. aeruginosa* for domestic birds [21].

In the nestlings examined here, bacterial transmission likely occurs horizontally, through direct penetration of the eggshell during the 10–14 days of incubation period typical for European goldfinches, or possibly shortly after hatching via maternal cloacal or oral microbes present in the nest, especially considering the environmental survival of both bacteria [21]. The fecal–oral route is generally considered the most likely transmission pathway for *Pseudomonas* spp., and it is also strongly suspected in *K. pneumoniae*-induced mortality in canaries [21,26]. It could be possible that adult goldfinches served as reservoirs for the pathogens identified in this study; however, it has not been investigated here and should be addressed in future studies. Moreover, considering the broad host range of both bacteria isolated here, the role of wild birds as a reservoir of infection cannot be excluded, as goldfinches were housed in open-air cages [51,52]. Indeed, several strains of *K. pneumoniae* are commonly isolated from pigeons and other free-roaming birds, as well as *P. aeruginosa*, which is part of the physiological pharyngeal and cloacal microbiota of some free-living birds [51].

Nestling finches in this study showed nonspecific clinical signs, such as depression, weakness, anorexia, abdominal distension and respiratory distress, and were found dead 1–4 days after hatching, while no clinical disease was observed in adults housed in the same cages. Certainly, clinical disease and the severity of bacterial infections are influenced by host susceptibility, immune response, and genetics and are dependent on nutritional status and management practices in captive passerines [4]. It is therefore not surprising that nestling birds with weakened immune systems may be more susceptible compared to clinically healthy, immunocompetent adult birds potentially harboring and transmitting potentially pathogenic microorganisms. Furthermore, clinically healthy adult passerines may harbor less virulent *K. pneumoniae* strains encoding fewer virulence factors than the more virulent strains associated with disease [23].

The main pathological findings in goldfinches examined in this study included severe, subacute bronchopneumonia and necrotizing hepatitis, with abundant aggregates of Gram-negative bacilli, especially in the parabronchial walls, in the lumen of the pulmonary arteries and surrounding hepatocellular necrotic foci. *K. pneumoniae* generally acts as a respiratory pathogen in birds, although it is sometimes responsible for gastrointestinal disease, with systemic spread and high mortality, especially in chicks, occurring under adverse conditions [15,21,22,23,26]. Necrotizing hepatitis with abundant intralesional bacilli has been observed in canary chicks naturally infected with *K. pneumoniae* of gastro-intestinal origin causing extraenteric disease and systemic lesions [26], similar to the hepatitis observed here. *P. aeruginosa* also causes respiratory tract infections in passerines, being responsible for pneumonia and airsacculitis, and has also been associated with necrotizing hepatitis in pet birds, a condition also associated with *Pseudomonas fluorescens* [21,53]. Of note, primary respiratory tract infections caused by *K. pneumoniae* and *P. aeruginosa* can progress to bacteriemia, primarily involving the liver [5]. Although less frequently, primary gastrointestinal tract infections can also result in bacteriemia, or less frequently, ascending infection to the liver via the biliary system [8]. However, in the finches analyzed here, considering the primary tropism of the pathogens isolated, it is likely that bacteriemia with severe hepatic involvement followed a primary co-infection of the respiratory tract. Since, with the exception of highly virulent bacteria, most cases of bacterial pneumonia in domestic birds are secondary to underlying viral diseases or other disorders [8], the presence of suspected viral pathogens was investigated. Specifically, based on clinical history and gross pathology (e.g., respiratory tract infection; necrotizing hepatitis; occasional interstitial nephritis), viral agents considered in the differential diagnosis included *New Castle Disease virus*, *Canary Circovirus*, *Canary Polyomavirus* and *Avian Bornavirus* [1,8,54,55]. However, no histopathological lesions suggestive of viral infections or the presence of viral inclusion bodies were observed, and finally, none of these viruses were isolated here by molecular investigations, although their presence was investigated in FFPE tissue samples.

In this study, we selected four housekeeping genes (16S rRNA, *rpoB*, *khe* and *ecfX*) for bacterial identification at species level [31,32]. Sequence analysis based only on the 16S rRNA gene is useful for distinguishing bacteria, but in most cases only allows bacterial classification up to the genus level, also in the case of *Klebsiella* and *Pseudomonas* species [30,31,56]. The *khe* and *rpoB* genes were further selected to identify *Klebsiella* strains at the species level [32,57]. The *khe* gene encodes a ‘unique’ hemolysin of *K. pneumoniae* and has been proposed as a unique gene, which is highly conserved among *K. pneumoniae* isolates [58]. However, comparative sequencing analyses of the 16S rRNA, *khe* and *rpoB* genes for the identification of *K. pneumoniae* reveal that the *rpoB* gene may enable improved identification of *K. pneumoniae* strains at the species or even subspecies level [32]. Of note, the *rpoB* gene emerged as a key candidate gene for confirming the identification of *K. pneumoniae*, thus allowing the discrimination of closely related isolates [57]. Regarding *P. aeruginosa*, *ecfX* PCR screening is species-specific and highly sensitive, not amplifying DNA from any of the other *Pseudomonas* species [31]. Indeed, the *ecfX* gene encodes an extracytoplasmic function (ECF) sigma factor restricted to *P. aeruginosa* and may play a role in heme uptake and virulence [31].

## 5. Conclusions

This study focused on the pathological findings of a co-infection with *K. pneumoniae* and *P. aeruginosa* as primary respiratory pathogens with bacteriemia in nestling goldfinches, leading to mortality during the first week after hatching. Although these Gram-negative pathogens are common in human bacterial pneumonia and often isolated simultaneously, to the authors’ knowledge, co-infection with these bacteria has not been previously documented in goldfinches. Pathogen identification is certainly essential for formulating a correct etiological diagnosis and further choosing an appropriate therapeutic strategy, by performing antibiotic susceptibility tests on isolated bacterial strains, and this should be addressed in future studies. Moreover, given the opportunistic behavior of these pathogens, which may be harbored by clinical healthy goldfinches, introducing new birds into flocks based solely on a clinical examination represents a great challenge.

## Figures and Tables

**Table 1:** Primers used in this study for bacteria identification [30,31,32].

Gene	Base Pairs	Forward Primer	Reverse Primer
16S rRNA	1500	AGAGTTTGATCMTGGCTCAG	TACGGYTACCTTGTTACGACTT
*ecfX*	500	ATGGATGAGCGCTTCCGTG	TCATCCTTCGCCTCCCTG
*khe*	430	TGATTGCATTCGCCACTGG	GGTCAACCCAACGATCCTG
*rpoB*	1000	GGCGAAATGGCWGAGAACCA	GAGTCTTCGAAGTTGTAACC

**Table 2:** Primers and Taqman probes used for real-time RT-PCR and conventional PCR. NDV: *NewCastle Disease Virus*; BORNA P: *Psittaciform 1 orthobornavirus*; BORNA C: passerine bornaviruses; CIRCO: *Circovirus*; VP1 POLY I/II: late gene encoding for the VP1 structural protein of *polyomavirus*.

NAME	SEQUENCE	FINAL CONCENTRATION	TYPE OF PCR PERFORMED
NDV F	GAGCTAATGAACATTCTTTC	0.5 µM	Real-Time PCR
NDV R	AATAGGCGGACCACATCTG	0.5 µM	
LPROMGB (PROBE)	FAM-CCAATCAACTTCCC-MGBEQ	0.2 µM	
LPROMGB2 (PROBE)	CY5-AATAGTGTATGACAACAC-MGBEQ	0.2 µM	
BORNA PCA3 (+) F	CCCGCAGACAGYACGT	0.25 µM	
BORNA PCA3 (+) F	GATCCGCAGACAGYACGT	0.25 µM	
BORNA PCA6 (-) R	AAGAAYCCNTCCATGATCTC	0.25 µM	
BORNAP (PROBE)	FAM-CGAATWCCCAGGGAGGCYCT-BHQ1	0.25 µM	
BORNAC (PROBE)	TexasRed-AGATGCATTGACCCARCCRGT-BHQ2	0.25 µM	
CIRCO-FOR	TTCACCCTTAAYAAYCCT	0.5 µM	Conventional PCR
CIRCO-REV	CCRTSATATCCATCCCACCA	0.5 µM	
VP1 POLY I FOR	CCAGACCCAACTARRAATGARAA	0.9 µM	
VP1 POLY I REV	AACAAGAGACACAAATNTTTCCNCC	0.9 µM	
VP1 POLY II FOR	ATGAAAATGGGGTTGGCCCNCTNTGYAARG	0.9 µM	
VP1 POLY II REV	CCCTCATAAACCCGAACYTCYTCHACYTG	0.9 µM	

## Data Availability

Not applicable.

## Supplementary Materials

The following supporting information can be downloaded at: https://www.mdpi.com/article/10.3390/vetsci12090821/s1, File S1: Representative 16S rRNA sequences submitted to GenBank.
