# Supplementary material for: Pathological Findings of Nestling European Goldfinches (Carduelis carduelis) Co-Infected with Klebsiella pneumoniae and Pseudomonas aeruginosa
Source: Vet Sci. 2025 Aug 27;12(9):821. doi: 10.3390/vetsci12090821 (PMC12474122; doi:10.3390/vetsci12090821)
Supplement: Supplementary file 1 [file vetsci-12-00821-s001.zip › vetsci-3774117-supplementary.pdf]

>Contig_COLONY.1_1A_1B(CR)_Trimmed
CCTAACACATGCAAGTCGAGCGGTAGCACAGAGAGCTTGCTCTCGGGTGACGAGCGGCGGACGGGTGAGTA
ATGTCTGGGAAACTGCCTGATGGAGGGGGATAACTACTGGAAACGGTAGCTAATACCGCATAATGTCGCAAG
ACCAAAGTGGGGGACCTTCGGGCCTCATGCCATCAGATGTGCCCAGATGGGATTAGCTAGTAGGTGGGGTAA
CGGCTCACCTAGGCGACGATCCCTAGCTGGTCTGAGAGGATGACCAGCCACACTGGAACTGAGACACGGTCC
AGACTCCTACGGGAGGCAGCAGTGGGGAATATTGCACAATGGGCGCAAGCCTGATGCAGCCATGCCGCGTG
TGTGAAGAAGGCCTTCGGGTTGTAAAGCACTTTCAGCGGGGAGGAAGGCGTTAAGGTTAATAACCTTGGCG
ATTGACGTTACCCGCAGAAGAAGCACCGGCTAACTCCGTGCCAGCAGCCGCGGTAATACGGAGGGTGCAAG
CGTTAATCGGAATTACTGGGCGTAAAGCGCACGCAGGCGGTCTGTCAAGTCGGATGTGAAATCCCCGGGCTC
AACCTGGGAACTGCATTCGAAACTGGCAGGCTAGAGTCTTGTAGAGGGGGGTAGAATTCCAGGTGTAGCGG
TGAAATGCGTAGAGATCTGGAGGAATACCGGTGGCGAAGGCGGCCCCCTGGACAAAGACTGACGCTCAGGT
GCGAAAGCGTGGGGAGCAAACAGGATTAGATACCCTGGTAGTCCACGCCGTAAACGATGTCGATTTGGAGGT
TGTGCCCTTGAGGCGTGGCTTCCGGAGCTAACGCGTTAAATCGACCGCCTGGGGAGTACGGCCGCAAGGTTA
AAACTCAAATGAATTGACGGGGGCCCGCACAAGCGGTGGAGCATGTGGTTTAATTCGATGCAACGCGAAGA
ACCTTACCTGGTCTTGTCTTCCACCACACATTCACGAGATGCATTGGTGCCTTCGGGAACTGTGAGACAGGTG
CTGCATGGCTGTCGTCAGCTG

Sequences producing significant alignments Download Select columns Show 100

select all *100 sequences selected* GenBank Graphics Distance tree of results MSA Viewer

Description	Scientific Name	Max Score	Total Score	Query Cover	E value	Per. Ident	Acc. Len	Accession
Klebsiella pneumoniae strain ZG2017CW4-3-1-2N chromosome, complete genome	Klebsiella pneum...	1842	14560	99%	0.0	99.22%	5355675	CP065351.1
Klebsiella sp. strain TA-T-1 16S ribosomal RNA gene, partial sequence	Klebsiella sp.	1840	1840	99%	0.0	99.12%	1480	OK326442.1
Klebsiella sp. strain BG-T-1 16S ribosomal RNA gene, partial sequence	Klebsiella sp.	1840	1840	99%	0.0	99.12%	1481	OK326033.1
Klebsiella pneumoniae strain ZY304 chromosome, complete genome	Klebsiella pneum...	1838	14586	99%	0.0	99.12%	5586931	CP158286.1
Klebsiella pneumoniae strain U-KP1402 chromosome, complete genome	Klebsiella pneum...	1838	14537	99%	0.0	99.12%	5362316	CP158302.1
Klebsiella pneumoniae strain K8 chromosome, complete genome	Klebsiella pneum...	1838	14497	99%	0.0	99.12%	5349345	CP126622.1
Klebsiella pneumoniae strain KP13 chromosome, complete genome	Klebsiella pneum...	1838	14569	99%	0.0	99.12%	5532312	CP154632.1
Klebsiella pneumoniae strain 413706 chromosome, complete genome	Klebsiella pneum...	1838	14558	99%	0.0	99.12%	5243682	CP154604.1
Klebsiella pneumoniae strain JXHV-KP2 chromosome, complete genome	Klebsiella pneum...	1838	14586	99%	0.0	99.12%	5473594	CP154343.1
Klebsiella pneumoniae strain JXLV-KP54 chromosome, complete genome	Klebsiella pneum...	1838	14586	99%	0.0	99.12%	5475614	CP154273.1
Klebsiella pneumoniae strain KP150 chromosome, complete genome	Klebsiella pneum...	1838	14586	99%	0.0	99.12%	5377447	CP154297.1
Klebsiella pneumoniae strain KP22 chromosome, complete genome	Klebsiella pneum...	1838	14569	99%	0.0	99.12%	5528946	CP154293.1
Klebsiella pneumoniae strain KP33 chromosome, complete genome	Klebsiella pneum...	1838	14586	99%	0.0	99.12%	5550846	CP154391.1
Klebsiella pneumoniae strain KP156 chromosome, complete genome	Klebsiella pneum...	1838	14586	99%	0.0	99.12%	5506082	CP154301.1

>Conting_COLONY.2_1C_1D(CR)_Trimmed
CGGCAGGCCTAACACATGCAAGTCGAGCGGATGAAGGGAGCTTGCTCCTGGATTCAGCGGCGGACGGGTGA
GTAATGCCTAGGAATCTGCCTGGTAGTGGGGGATAACGTCCGGAAACGGGCGCTAATACCGCATACGTCCTGA
GGGAGAAAGTGGGGGATCTTCGGACCTCACGCTATCAGATGAGCCTAGGTCGGATTAGCTAGTTGGTGGGGT
AAAGGCCTACCAAGGCGACGATCCGTAACTGGTCTGAGAGGATGATCAGTCACACTGGAACTGAGACACGGT
CCAGACTCCTACGGGAGGCAGCAGTGGGGAATATTGGACAATGGGCGAAAGCCTGATCCAGCCATGCCGCG
TGTGTGAAGAAGGTCTTCGGATTGTAAAGCACTTTAAGTTGGGAGGAAGGGCAGTAAGTTAATACCTTGCTG
TTTTGACGTTACCAACAGAATAAGCACCGGCTAACTTCGTGCCAGCAGCCGCGGTAATACGAAGGGTGCAAG
CGTTAATCGGAATTACTGGGCGTAAAGCGCGCGTAGGTGGTTCAGCAAGTTGGATGTGAAATCCCCGGGCTC
AACCTGGGAACTGCATCCAAAACTACTGAGCTAGAGTACGGTAGAGGGTGGTGGAATTTCCTGTGTAGCGGT
GAAATGCGTAGATATAGGAAGGAACACCAGTGGCGAAGGCGACCACCTGGACTGATACTGACACTGAGGTG
CGAAAGCGTGGGGAGCAAACAGGATTAGATACCCTGGTAGTCCACGCCGTAACGATGTCGACTAGCCGTTGG
GATCCTTGAGATCTTAGTGGCGCAGCTAACGCGATAAGTCGACCGCCTGGGGAGTACGGCCGCAAGGTTAAA
ACTCAATTGAATTGACGGGGGCCCGCACAAGCGGTGGAGCATGTGGTTTAATTCGAAGCAACGCGAAGAAC
CTTACCTGGCCTTGACATGCTGAGAACTTTCCAGAGATGGATTGGTGCCTTCGGGAACTCAGACACAGGTGCT
GCAATGGCTGTCGTCAGCTCGTGTC

Sequences producing significant alignments Download Select columns Show 100

Pseudomonas aeruginosa

Select all 100 sequences selected GenBank Graphics Distance tree of results MSA Viewer

	Description	Scientific Name	Max Score	Total Score	Query Cover	E value	Per. Ident	Acc. Len	Accession
☑	Pseudomonas aeruginosa strain R8-770-1 16S ribosomal RNA gene, partial seq…	Pseudomonas aerugi…	1893	1893	100%	0.0	99.81%	1354	JQ659984.1
☑	Pseudomonas aeruginosa strain R8-770 16S ribosomal RNA gene, partial seque…	Pseudomonas aerugi…	1893	1893	100%	0.0	99.81%	1370	JQ659983.1
☑	Pseudomonas sp. strain 2B 16S ribosomal RNA gene, partial sequence	Pseudomonas sp.	1888	1888	100%	0.0	99.71%	1503	PP859489.1
☑	Pseudomonas aeruginosa strain PA_HN006 chromosome, complete genome	Pseudomonas aerugi…	1888	7553	100%	0.0	99.71%	6802378	CP113235.1
☑	Pseudomonas aeruginosa strain PA_HN005 chromosome, complete genome	Pseudomonas aerugi…	1888	7553	100%	0.0	99.71%	6795494	CP118566.1
☑	Pseudomonas aeruginosa isolate PA_HN008 chromosome, complete genome	Pseudomonas aerugi…	1888	7553	100%	0.0	99.71%	6591441	CP118564.1
☑	Pseudomonas aeruginosa strain PA_HN004 chromosome, complete genome	Pseudomonas aerugi…	1888	7553	100%	0.0	99.71%	6447198	CP118565.1
☑	Pseudomonas aeruginosa strain CUVET23-830 chromosome, complete genome	Pseudomonas aerugi…	1888	7553	100%	0.0	99.71%	6922301	CP130957.1
☑	Pseudomonas aeruginosa strain PAE3 chromosome, complete genome	Pseudomonas aerugi…	1888	7553	100%	0.0	99.71%	7073555	CP154349.1
☑	Pseudomonas aeruginosa Kenaga1 DNA, nearly complete genome	Pseudomonas aerugi…	1888	7553	100%	0.0	99.71%	6375372	AP031457.1
☑	Pseudomonas aeruginosa strain NCTR 501 chromosome, complete genome	Pseudomonas aerugi…	1888	7553	100%	0.0	99.71%	6420288	CP152075.1
☑	Pseudomonas aeruginosa strain ZYPA162 chromosome, complete genome	Pseudomonas aerugi…	1888	7553	100%	0.0	99.71%	6591652	CP132997.1
☑	Pseudomonas aeruginosa strain PGI3 16S ribosomal RNA gene, partial sequence	Pseudomonas aerugi…	1888	1888	100%	0.0	99.71%	1474	OQ851991.1
☑	Pseudomonas aeruginosa strain PS03095 chromosome, complete genome	Pseudomonas aerugi…	1888	7553	100%	0.0	99.71%	6541669	CP152373.1
☑	Pseudomonas aeruginosa strain 2572 chromosome	Pseudomonas aerugi…	1888	6971	100%	0.0	99.71%	6264677	CP138510.1
